# What Do We Mean by Internet Access? A Framework for Health Researchers

**Published:** 2004-09-15

**Authors:** Nigel E. Bush, Deborah J Bowen, Jean Wooldridge, Abi Ludwig, Robert Robbins, Hendrika Meischke

**Affiliations:** Fred Hutchinson Cancer Research Center; Fred Hutchinson Cancer Research Center, Seattle, Washh; Fred Hutchinson Cancer Research Center, Seattle, Wash; Fred Hutchinson Cancer Research Center, Seattle, Wash; Fred Hutchinson Cancer Research Center, Seattle, Washh; University of Washington, Seattle, Wash

## Abstract

Much is written about Internet access, Web access, Web site accessibility, and access to online health information. The term *access* has, however, a variety of meanings to authors in different contexts when applied to the Internet, the Web, and interactive health communication. We have summarized those varied uses and definitions and consolidated them into a framework that defines Internet and Web access issues for health researchers. We group issues into two categories: connectivity and human interface. Our focus is to conceptualize access as a multicomponent issue that can either reduce or enhance the public health utility of electronic communications.

## Introduction

The Internet and World Wide Web (Web) have rapidly become ubiquitous in the lives of the majority of Americans. By March 2004, three fourths of Americans were able to use the Internet from home (1). Americans routinely turn to the Web for information, entertainment, merchandise, and communication. In particular, the Internet has become a prime source of health information for consumers (2,3). Although the Internet has potential as a tool for health improvement, its impact hinges on issues of access. Access is an issue that affects people at home, at school, and in the community at large (4). Even where access to basic Internet infrastructure exists or is provided, further access to Internet use is often limited by other factors (5). A more global concept of Internet access encompasses a spectrum of narrower, interrelated factors described by Eng et al as "the ability to access, comprehend, and utilize information and support appropriate to one's personal characteristics" (6).

In this article, we document the myriad uses and definitions of Internet access from a wide variety of sources and consolidate them into a single, comprehensive, cohesive framework suitable for health research and practice. We believe the proposed framework will provide researchers a clearer and more thorough understanding of Internet access, whether they design Web-based interventions, implement electronic outcomes assessments, develop online educational resources, or otherwise incorporate interactive health communications (IHC) components in their research endeavors.

### Need for clarifying and specifying terms

Our own research experience illustrates the difficulties of the issues of access. We recently conducted pilot research on communicating breast cancer risk to low-income, predominantly African American, elderly, inner-city women. As part of this research, we tested a computer and Internet education and training program in local church community centers among a sample of our target audience. One of our first steps was to identify a group of Web *nonusers*, or novices, through telephone questionnaires. We had to revise our initial questionnaire several times before we were able to define and isolate our target group. Who exactly was a nonuser? Was a nonuser someone who had never used the Internet or Web, someone who may have used it occasionally but not recently, or someone who used it frequently but stopped? What did we mean by occasionally or recently? What degree of use determined a user? Did occasional e-mailing with help from another person constitute Internet experience? Should we include women who were computer users but who had little or no Internet experience? Was a nonuser also someone who had no access to an Internet connection? By "no access" did we mean no easy or convenient access? If so, how did we define ease or convenience and what was our cut-off criterion? Was it availability in the home only or availability within easy walking distance from the home (e.g., church, community center)? The permutations were endless.

The details of our final framework are based both on our own experience and on the findings of other Web-based health research projects (7,8) and are described below. 

## Methods

### Distinguishing between the Internet and the Web

Within the technology community, a clear demarcation exists between the Internet and the Web. In common usage, however, the two are often confused, with Internet and Web frequently used interchangeably as if they were the same entity. Motive, a New Zealand-based Internet communication design company, defines the Internet in relatively lay terms as "a global network of interconnected computers. This is the infrastructure through which applications such as e-mail, chat rooms and instant messaging operate" (9). Motive goes on to distinguish the Internet from the Web: "Thus, the Web is an example of an Internet application. The Web is accessed through a browser which can display text, images, and time-based media and allow a user to access applications" (9). December Communications, a Web-based communications company, reminds us in more technical terms that "[t]he Web is not the Internet itself. The Web is not a proprietary system like AOL. Instead, the Web is a system of clients (Web browsers) and servers that uses the Internet for its data exchange" (10). Foldoc, an online dictionary of computing, similarly describes a Web browser: "The client program (known as a browser), e.g., Netscape Navigator, runs on the user's computer and provides two basic navigation operations: to follow a link or to send a query to a server" (11).

### Information source

We explored three overlapping information sources that cover the various uses of Internet and Web access: 1) the academic, medical, and health literature, 2) the Web itself, including recent Internet usage surveys, and 3) seminal reports on eHealth and online health consumers. Searches of Medline and PubMed for the words "Internet" and "Web" in any field each yielded more than 3000 articles, and more than 1500 hits resulted when we combined the two search terms. Searches for "World Wide Web" produced more than 500 results. These three searches produced results that were beyond the scope of this summary paper, so we then narrowed our search to articles published in the last five years with the words "Internet" or "Web and Access" in their titles and abstracts. This more focused search generated a more wieldy 200 articles. We also searched the Web for definitions and examples of Web access, Internet access, and variations on those themes in the commercial and private sector using search engines such as Google (12) and WebFerret (13). In addition, we reviewed recent Internet communication and survey sites including the Pew Internet and American Life Project (14), Nielsen//NetRatings (15), Harris Interactive (16), and Nua Internet Surveys (17). Finally, for definitions, uses, and terminology, we inspected a selection of recent seminal reports on e-health, online health, and the "digital divide," including *Healthy People 2010* (18), *The eHealth Landscape* (5), *Wired for Health* (19), *A Nation Online* (20), *Falling through the Net* (21), and *The UCLA Internet Report* (22).

## Results

### A health researcher's framework of Internet and Web access

**Figure 1:**
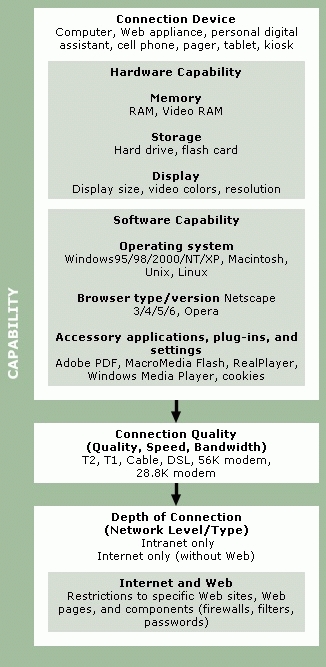

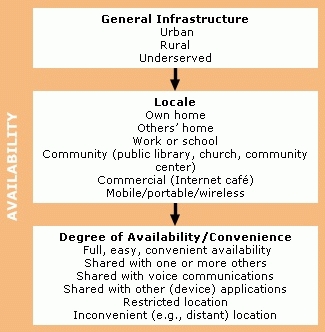
Examples of elements of connectivity, which is defined as “connecting or being connected to the Internet, the Web, a Web site, Web page, or Web subcomponent; having the functionality and content of the Internet and/or Web physically available.” The categories above provide noninclusive lists of examples.

**Figure 2:**
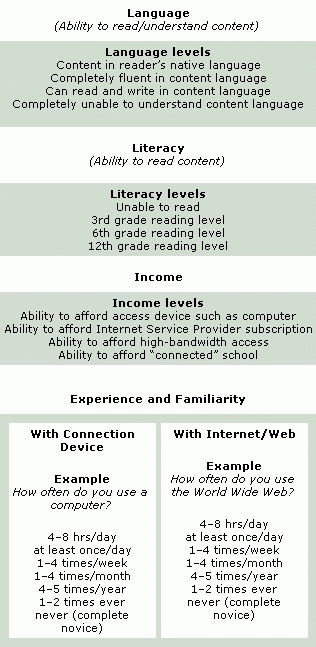

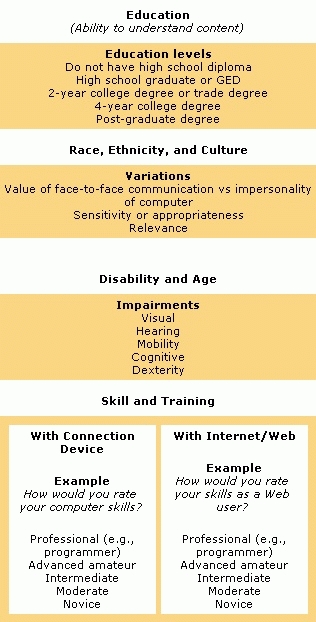
Examples of elements of human interface, which is defined as “those factors relating to user demographics and characteristics, such as literacy, language, education, race, ethnicity and culture, income, disability and age, experience and familiarity, and skill and training, which determine or restrict level of access to Internet/Web and content.” The categories above provide noninclusive lists of examples.

Figures 1 and 2 present our proposed framework for describing Internet and Web access. In the process of consolidating results from our search of the literature, Web, and other sources, we sorted the disparate and varied uses and definitions of access into coherent unifying clusters, or collective grouping, based on similar meanings and usages. Initially, we created a relatively large number of small clusters, with individual examples often allocated to more than one cluster. We then progressively combined clusters that we judged to share similar overriding characteristics into fewer, more broadly descriptive and exclusive groupings. Finally, we assigned our final clusters to one of two global categories. We propose that issues of Internet and Web access can be catalogued as either connectivity (Figure 1) or human interface (Figure 2) issues. These global categories are not mutually exclusive, and many of the examples within each category interact and co-vary with others to different extents; we believe, however, the two global categories offer a simple and convenient descriptive framework.

### Connectivity

We broadly define *connectivity* as "connecting or being connected to the Internet, the Web, a Web site, Web page, or Web subcomponent; having the functionality and content of the Internet and/or Web physically available." A number of sources in the academic literature describe access simply as being connected to the Internet or Web (23-54). We found, for example, references to free Internet access (49), access to the Internet during dentist visits (55), Internet access through an employer (41), use of touch-screen kiosks to provide Internet access (56,57), degree or quality of connectivity (e.g., Broadband service) (24), and common places of access, such as home (58,59) or work (43). Predominantly commercial Web sites and Web surveys also focused primarily on this kind of basic connectivity. SearchVB.com described access in this way: "Web access means having a connection to the World Wide Web through an access provider or an online service provider such as America Online" (60). In addition, theDirectory.org described Internet access providers as "companies that provide connections to the Internet for businesses and individuals" (61). Nielsen//NetRatings Audience Measurement Service reports Internet usage estimates based on a sample of households that have access to the Internet. The Nielsen//NetRatings Internet universe is defined as all members of U.S. households (aged two years or older) that currently have access to the Internet (62). Harris Interactive seems to define access as "computer users who are online." For example, "Two-thirds (66%) of all adults are now online. This includes more than half (55%) of all adults who access the Internet from home, almost a third (30%) who access it from work, and almost one in five adults who go online from a school, library, cyber café or other location" (63). In addition, Systems Computing Services distinguishes between connection and access: "When connected to the Internet, you have access to several kinds of resources" (64).

Glossary

Browser: a software application used to find and display Web pages.
Kiosk: a stand-alone booth providing a computer-related service. Kiosks must be easy to use (without training or documentation), and the hardware must be capable of operating unattended for long periods of time. Examples of kiosks include automated teller machines and tourist information booths.
Firewall: a barrier designed to protect a private network from unauthorized access. Information going through the firewall in either direction is examined to make sure it meets security criteria. Firewalls can be implemented in hardware, software, or both.
Plug-in: a piece of hardware or software that adds a specific feature or service to a larger system. The idea is that the new component simply plugs into the existing system, but it must be installed separately from the existing system.
Intranet: a private network that operates like the Internet but is accessible only to a limited group of users, such as a company’s employees. Many intranets are also connected to the Internet, but they are protected by a firewall.
Functionality: what the features of hardware or software enable a user to do.
Application: software applications are the programs (or groups of programs) that enable users to accomplish tasks. Examples include word processing and spreadsheet programs as well as e-mail programs and Web browsers.
Networking: connecting two or more computers together so they can communicate with each other.
Bandwidth: the amount of data (pieces of information) that can be transmitted in a fixed amount of time.
Coding: writing the instructions for a computer program. There are many different types of code as well as computer languages in which they can be written.
RAM: (Random Access Memory) — the type of memory, or data storage, used for storing data temporarily while working on a computer. RAM is volatile, which means that when the power is turned off, the contents stored in RAM are lost. Computer memory can be thought of as boxes, each of which holds a single byte of information. If a computer has 8MB RAM, then eight million bytes of memory are available for programs to use.

Other varied references to access as basic connectivity included:

- "Internet access from home: To reach the Internet the user needs service from 1) a communications company (i.e., a telephone, cable television, or wireless company)"Internet access from home: To reach the Internet the user needs service from 1) a communications company (i.e., a telephone, cable television, or wireless company) providing a transport service to physically transmit data to and from the consumer's home and 2) an ISP [Internet Service Provider] providing access to the Internet" (65). 
- "80 percent of Americans access the Internet through dial-up service," and "Internet access is more frequently occurring outside the home, at such locations [defined] as work, school, public libraries, community centers, someone else's house, and somewhere else" (20).
- "Americans bought home computers and hooked them up to the Internet at a remarkable rate between December 1998 and August 2000. Virtually every group has participated in the sharp upward trend of Americans connecting their homes to the Internet" (66). 
- "Interactive health communication (IHC): the interaction of an individual — consumer, patient, caregiver, or professional — with or through an electronic device or communication technology to access or transmit health information, or to receive or provide guidance and support on a health-related issue" (19). 

The majority of sources in the academic literature also describe Web or Internet access as opening, using, or getting to content, documents, and applications and collecting data (31,36,37,48,67–110). This description is necessarily broad and encompasses a variety of types of access and types of content accessed. General examples include access to patient/medical records and clinical information (111–125); a variety of online databases (126–148), including blood bank (149) and sperm bank (150) information; teaching and education syllabi (151,152); other computer systems (153,154), such as libraries (155); continuously acquired physiological patient data (156) or real-time diagnoses (157) by physicians; medical expertise (88,158,159); online patient decision-support tool (160); specific, sometimes difficult-to-find populations (75); and populations for online surveys (161).

*Availability*. We first qualify connectivity in terms of its availability. The location and availability of the connection device are important in determining degree, ease, and convenience of access. For example, we may describe a group of users as having home Internet access because a survey tells us that each individual within the group has a home computer connected by telephone modem to an ISP. The single question, "Do you have a computer at home connected by telephone modem to an ISP?," however, tells us little about the availability of Internet access. In one home, a single occupant may be the sole user of the Internet connection. In another home, availability of the home Internet connection may be much more restricted — use may be shared, regulated, severely limited or even denied, perhaps by some other person in the house. Both examples depict home Internet access but vary considerably in degree.

Also critical in determining access is the availability of local ISPs (not requiring a long-distance call), and, more frequently, adequate quality of connection (bandwidth and choice of medium). Both factors vary greatly with geography, especially between urban and rural areas. For example, a potential user in a more remote rural area simply may not have available high-speed Internet service (21,162).

The location of the connection device must also be considered when defining availability of access. Connections to the Internet are commonly made from the home, from work or school, or from local communal points such as community centers, church halls, public libraries, or Internet café Internet users without home connections may also connect from other people's homes, an option often forgotten in access surveys. And the Internet now can be reached via mobile or portable devices without fixed locations.

Connection at and between each location varies in the degree of availability, convenience, and ease of access. Availability might be restricted because of multiple users (i.e., the obligation to share the connection with one or more other users). Availability might also be restricted if the connection uses the only telephone line available at that location (i.e., sharing a voice line). At work, the connection device might be located in an office with restricted physical access. At school, the device might be in a computer lab with availability rigorously scheduled (i.e., limited times and hours). Finally, the connection location itself might be inconvenient. In some urban areas, a connection location outside of the home, work, or school can often be found within a few blocks; in other locales, the nearest place to go online may be preclusively distant.

The implication of describing Web site availability is that some restriction may prevent users from opening or using the site. For example, describing a Web site as "publicly accessible" (163) implies that other sites may not be accessible by the general public but are limited to designated users; security measures may be employed to limit access to a site or to specific site content (164) or to a computer system (154). One of the most common examples in this context is the privileged (restricted to authorized users only) access to medical records (90,113,121,122,125,141,165). Alternately, a site, or information within it, may be inaccessible because of design or coding issues; for example, specific content or sites may be inaccessible to search engines (166,167). Access and use also may be hindered by navigational challenges due to numerous design features (e.g., disorganization, technical language, lack of permanence [70], or simple download time [168]).

*Capability*. We further qualify connectivity in terms of capability. The capability (and configuration) of both hardware and software determines how efficiently the content and functionality of the Internet or Web is accessed and how comprehensively the content and functionality are made available. With lesser capability, some content or functionality will not be accessible or available.

Hardware capability and configuration can determine how much of a Web page is visible, the quality or resolution of that view, or how many Web pages can be opened at one time. For example, a PDA (personal digital assistant) with 8MB (megabytes) of RAM and small monochrome display is considerably less capable of opening, displaying, and manipulating a typical Web page than is a late-model desktop computer. Hardware capability also determines to some extent software capability. More powerful and fully featured software applications typically require more RAM, larger hard-drive storage capacity, and faster computer processing speed and power to function optimally. Less capable hardware can diminish software performance. Additionally, Internet and Web content and functionality may be optimized for, or even require, specific versions and types of software. For example, many multimedia Web pages can only be optimally opened, viewed, and manipulated using a recent-version Web browser of a specific brand and third-party software plug-ins.

The capability of the connection between the access device, such as a computer, and the Internet and/or Web is also critically important to overall access. Most home Internet users connect from a desktop computer via modem and standard telephone line to an ISP. An increasing number of users connect via faster telephone connections called DSL (digital subscriber line), which allows data transmission without interfering with telephone voice service, while a few home users and many people at work use much faster dedicated cable or T1 lines. The bandwidth of the connection and/or the speed of the modem determine how quickly Web content and other data download from the Internet to the user device. For example, a Web page containing large files such as graphic elements or audio-video features, because of prohibitive download times, may be largely inaccessible to a user with a slow telephone connection.

We distinguished previously between the Internet and the Web. Some Internet users do not connect to or use the Web; instead, they use non-Web networking to access and transmit data. Examples include Pine^®^, an electronic messaging program that does not use the Web, but connects to the Internet, and FTP (file transfer protocol), which allows users to transfer files over the Internet without using the Web. Internet users may also use private or proprietary sets of networked tools and they may share applications. These users may have Internet access, but not Web access. Most home consumers, however, do connect to the Internet and to the special Internet application known as the Web. There are, therefore, varying degrees of network connectivity, including 1) intranets shared by or accessible to only a limited groups of users, 2) restricted or relatively unrestricted use of the Internet, and 3) the Web. We can specify degree of network connectivity more precisely by assessing availability of specific Web sites or even components of Web pages. Connections to both the Internet and the Web and their various components frequently are restricted by firewalls, ISP limitations and policies, content filters, passwords, and other boundaries. Availability of some Web sites may also be limited by their obscurity to search engines. Thus, Internet or Web access is related to the type and degree of network availability. When we say someone has "full Web access," we mean it only in the most generic terms. We assume that the user has functionality as well as availability to general Web content, but we also presume that specific sites and content are unavailable on a case-by-case basis or by type (e.g., pornography filtered by ISP or public library). 

### Human interface

We define the *human interface* category of Internet access as "those factors relating to user demographics and characteristics, such as literacy, language, education, race, ethnicity and culture, income, disability and age, experience and familiarity, and skill and training, which determine or restrict level of access to Internet/Web and content" (5,169). Again, many of these factors are not mutually exclusive but interact and covary with each other. Not surprisingly, our various sources contained frequent references to the relationships among Internet/Web access, health disparities, and individual, personal, or demographic limitations — the digital divide. 

To some extent, our human interface factors encompass, but are not confined to, issues commonly considered when assessing usability. Usability of a product or application typically refers to the quality of a user experience when interacting with the product or application, with an emphasis on behavior rather than opinion or recollection. Usability measures learnability, memorability, efficiency, frequency and severity of errors, and user satisfaction. Having evolved from observational methodology and ergonomics, the study of Web site usability has focused increasingly on human limitations, such as disability and literacy (170–174). We list and describe below human interface accessibility factors.

*Literacy*. For the content of a Web site to be accessible, it must be readable. A health-related Web site written at a college-graduate–level of literacy is inaccessible to a reader with a sixth-grade reading level  (168,175–178).

*Language*. The ability to read content is also determined by the user's language skills. A site written in English obviously is inaccessible to a monolingual native-French speaker, however rudimentary the written literacy level (48,168).

*Education*. For the content of a Web site to be accessible, it must also be understandable once it is read. We suggest that educational level may be the closest analog of the ability to understand information, especially health-related material (179).

*Race, ethnicity, and culture*. The content of a Web site may be both readable and understandable to a user, but at the same time it could also be culturally or ethnically insensitive, inappropriate, or irrelevant to the user and, therefore, relatively inaccessible. For example, a cancer-prevention–related Web site might illustrate quite vividly a cervical screening procedure that white individuals may deem acceptable, but that other readers (e.g., women with a traditional southeast Asian background) might find offensively candid (4,5,48,162,178,180).

*Income*. Income appears to predict Internet access even more than race and ethnicity (39). People of lower income are less likely to be able to afford either a home Internet connection device such as a computer or the regular subscription costs to an ISP. Lower-income people who connect to the Internet from home are less likely to afford a higher (faster) bandwidth connection or live in an area where it is available. Although other avenues of access are available in the community, they are less convenient than the home and, consequently, less often used. And the workplaces and schools of lower-income people are less likely to provide Internet connectivity (38-40,48,181-183).

*Disability and age*. We take for granted many of the skills and abilities necessary to access the Internet. We turn on a computer and manipulate a pointing device such as a mouse to open a connection to an ISP. We recall our private password and user name, type a Web address on the keyboard, and open a Web page. We read the text, look at the images, perhaps listen to audio; these tasks are denied to users with certain disabilities. And while these disabilities may be due to non-age–related causes, they most commonly are associated with advancing age. Thus, physical disability might restrict mobility (reaching the computer) or dexterity (accurate or speedy use of keyboard and mouse). Visual impairments such as myopia or color-blindness affect easy reading of text, which may vary in font size or color, or viewing of images. Hearing deficiency further restricts access to multimedia. Cognitive disability such as problems with memory and concentration limit the effectiveness of training, recall of passwords and educational content, navigation, and so on (21,170,184).

*Experience and familiarity*. A primary factor determining the level or degree of access to Internet and Web content and functionality is the user experience and familiarity with all the various aspects of connecting to the Internet and Web and navigating, manipulating, and otherwise using the Internet and Web once connected. We further distinguish between experience and familiarity with the connection device, usually a computer, and experience and familiarity with the Internet and Web once connected. By experience and familiarity, we mean how often and for what duration the individual has been exposed, either by personal use or vicariously, to the device and the Internet. Device experience and Internet experience are frequently but not necessarily related. A computer novice is unlikely to be an experienced Internet user; however, an individual may be a relatively experienced computer user but quite unfamiliar with the Web and largely unable to avail himself or herself of its features (18,48,182–185).

*Skill and training*. The issue of perception of skill often, but not necessarily, overlaps with experience and familiarity in affecting levels of Internet access. Our own anecdotal evidence suggests that some individuals may report considerable computer experience but judge themselves to be only moderately computer literate or skillful. We believe that technical knowledge and skills determine to some extent the degree of access to the Internet and Web. For example, a good working knowledge of computer and Web applications might better enable routine maintenance of the connection device or the installation of third party plug-in software when required for Web site access. Again, we relate skill separately to the connection device and to the Internet and Web. 

### Applying the framework

In our introduction, we described briefly our recent pilot research on communicating breast cancer risk. The initial difficulty in defining nonuser in our screening questionnaire was one of the factors that stimulated the writing of this paper and the development of our framework. Subsequently, we applied the framework as a guide to designing our final project procedures and the breast cancer risk Web site itself. Our target audience was low-income, predominantly African American, elderly, inner-city women in Seattle, Wash, who, to be eligible, had no Internet access and who were computer and Internet novices. Referring to our framework in our approach phone calls and screening questionnaires, we first considered *connectivity*. Because we were looking for nonusers, the capability of any connectivity was less relevant than *availability, *and we chose the general infrastructure of our target location to be urban-underserved and therefore unlikely to be "wired."  We first ascertained that each candidate had no home or convenient local availability of a computer or Internet connection (*locale* and *degree of availability*). We then turned to *human interface* factors and concurrently determined that each candidate had little or no *experience and familiarity* or *skill* with computers and the Web. We ensured that each candidate could minimally read and write in English (*language*). We then tailored our church-based training program and test Web site functionality and content to participant *literacy*, *education*, *race/ethnicity/culture*, and *disability/age*.

## Discussion

This paper has focused exclusively on a discussion of access, including access to the Internet and Web, content accessibility, and restrictions to access. We are well aware that the complexities of IHC go beyond mere access; they include the countless ways people use, interact with, and potentially benefit from new media. Established models of information processing, such as cognitive style preferences for perceiving, remembering, organizing, processing, thinking, and problem solving (186), are being newly applied to emerging technology applications such as the Web but are beyond the scope of this paper. In future papers, we will delve deeper into the intricacies of usability, learning style, and other issues (170-174).

Before we conclude, however, there is one factor relating to access that is often ignored: the possibility that many people do not use the Internet not because they lack access but simply because they do not want to use it or do not see a need to use it. This has serious implications for health care infrastructure spending, especially among the underserved. Current efforts on the digital divide have focused largely on providing access to computers and the Internet and to hardware and software training. One of the most popular access enhancement models is the establishment of community computer/Internet centers in lower-income neighborhoods, which have been supported by various foundations, corporations, local businesses, and government agencies (5). Yet despite gains in computer and Internet access reported early in 2002 by the Department of Commerce (20), a significant divide continues based on income, education and literacy, race and ethnicity, age, gender, geography, and disability (177,187,188). What remains unclear is to what extent the divide is due to poor access to information technology and how much it is due to low adoption of the technology where access exists. Conventional wisdom suggests that disparities in Internet use emanate from inequalities in infrastructure access, primarily in connectivity, and that providing access to the underserved alleviates the inequality. However, in many cultures, computers are simply not valued and may be resisted as poor alternatives to face-to-face communication (177). In our own city (Seattle), 82% of residents have access to the Internet, and yet adoption or use remains low, especially in some underserved communities (187). Although there are still local disparities in access, apparent lack of interest or perceived need is often cited as one of the highest barriers to Internet use (22,95), which may be mediated by ignorance of what the Internet has to offer (8).

We have described in significant detail a range of definitions for, myriad determinants of, and restrictions to Internet and Web access. We do not claim to have constructed the definitive taxonomy; in fact, that may be a futile goal given the rapid and unpredictable progress of IHC. We hope our efforts may, however, make health researchers more aware of the need for specificity and consistency in their reporting of Internet access-related topics and provide them with some choices.
